# Development and implementation of a geographical area categorisation method with targeted performance indicators for nationwide EMS in Finland

**DOI:** 10.1186/s13049-018-0506-1

**Published:** 2018-05-15

**Authors:** Jukka Pappinen, Päivi Laukkanen-Nevala, Pekka Mäntyselkä, Jouni Kurola

**Affiliations:** 1 FinnHEMS Research and Development Unit, Lentäjäntie 3, FI-01530 Vantaa, Finland; 2 University of Eastern Finland, Faculty of Health Sciences, P.O. Box 1627, FI-70211 Kuopio, Finland; 3 University of Eastern Finland, School of Medicine, P.O. Box 1627, FI-70211 Kuopio, Finland; 4 Primary Health Care Unit, Kuopio University Hospital, Kuopio, Finland; 5 Centre for Pre-hospital Emergency Care, Kuopio University Hospital, P.O. Box 1777, FI-70210 Kuopio, Finland

**Keywords:** Emergency medical services, Geographic information systems

## Abstract

**Background:**

In Finland, hospital districts (HD) are required by law to determine the level and availability of Emergency Medical Services (EMS) for each 1-km^2^ sized area (cell) within their administrative area. The cells are currently categorised into five risk categories based on the predicted number of missions. Methodological defects and insufficient instructions have led to incomparability between EMS services.

The aim of this study was to describe a new, nationwide method for categorising the cells, analyse EMS response time data and describe possible differences in mission profiles between the new risk category areas.

**Methods:**

National databases of EMS missions, population and buildings were combined with an existing nationwide 1-km^2^ hexagon-shaped cell grid. The cells were categorised into four groups, based on the Finnish Environment Institute’s (FEI) national definition of urban and rural areas, population and historical EMS mission density within each cell.

The EMS mission profiles of the cell categories were compared using risk ratios with confidence intervals in 12 mission groups.

**Results:**

In total, 87.3% of the population lives and 87.5% of missions took place in core or other urban areas, which covered only 4.7% of the HDs’ surface area.

Trauma mission incidence per 1000 inhabitants was higher in core urban areas (42.2) than in other urban (24.2) or dispersed settlement areas (24.6). The results were similar for non-trauma missions (134.8, 93.2 and 92.2, respectively).

Each cell category had a characteristic mission profile. High-energy trauma missions and cardiac problems were more common in rural and uninhabited cells, while violence, intoxication and non-specific problems dominated in urban areas.

**Conclusion:**

The proposed area categories and grid-based data collection appear to be a useful method for evaluating EMS demand and availability in different parts of the country for statistical purposes. Due to a similar rural/urban area definition, the method might also be usable for comparison between the Nordic countries.

**Electronic supplementary material:**

The online version of this article (10.1186/s13049-018-0506-1) contains supplementary material, which is available to authorized users.

## Background

Finland is divided into 20 hospital districts (HD), excluding the autonomous province of Åland. HDs are joint municipal authorities responsible for organising secondary care. Five of these are university hospital districts with additional responsibilities regarding EMS, e.g. Helicopter Emergency Medical Services (HEMS), as well as organisation of tertiary care. Since 2013, HDs have been responsible for organising Emergency Medical Services (EMS) within their boundaries.

Developing systematic performance indicators for EMS has been an ongoing issue for decades. Many EMS systems use response time as their primary quality indicator, despite criticism and observed unintended adverse effects [1, 2].

Since 2013, legislation has required HDs to make a formal decision on the availability and level of EMS service within their administrative area. Availability is measured by the percentage of missions reached within 8, 15, 30 and 120 min, depending on mission urgency and location (Table 1).

**Table 1:** Target percentages of 1-km^2^ cells by mission urgency and time limits as an example of EMS level of service (Pirkanmaa Hospital District service level target 2017–2018)

	A/B (lights&siren)			C (urgent)	D (non-urgent)
Cell risk category	1st EMS unit^a^within 8 min, %	1st EMS unit^a^within 15 min, %	ALS unit^b^within 30 min, %	Any ambulance within 30 min, %	Any ambulance within 120 min, %
1	85	95	95	95	95
2	65	95	95	90	95
3	45	80	95	85	95
4	20	60	90	80	95
5	Not defined				

^a^first responder of ambulance

^b^advanced life support unit

Until the end of 2017, the geographical risk classification was based on 1-km^2^ sized areas (cells) that were classified into five risk categories depending on the predicted number of EMS missions in a one-year period. Categories 1–4 had yearly mission limits of > 365, 365–52, 51–12 and < 12, respectively. Category 5 contained cells without permanent habitation or road access. Obviously, the category limits were chosen to comply with everyday calendar units. The method was loosely based on the one used for EMS in Nova Scotia, Canada [3].

To ensure equity and equality in EMS availability, each HD had to organise services so that areas belonging to the same risk category were reached with a similar level of service. HDs had to decide a percentage of missions reached within an 8-, 15-, 30- and 120-min timeframes. The Emergency Response Center classifies missions into four urgency classes (A–D), A and B being lights & siren, C urgent but without lights & siren and D being non-urgent [4].

HDs had to observe actualised response times and report twice a year to regional and national health supervisory authorities.

Over the years several flaws and defects have been observed in the current risk category system. Legislation did not define the method to predict the number of missions, nor the form of geographical cell grid. At least four different prediction methods or models and two different geographic grids were used [5]. This led to incomparability between HD data and reports, thus making it difficult for supervisory authorities to evaluate the equity and equality of EMS availability between HDs.

Many HDs have been applying linear regression models—with the populations in various age groups as explanatory variables—to predict the number of missions. However, the population is unevenly distributed between cells, and concentration of the population in urban areas has been an accelerating trend over the last decade, thus making linear regression predictions unrealistic for practical purposes.

Due to a wide range of values in current risk category 2, it contains areas from small town centres to rural hamlets. This has led to one-cell hotspots surrounded by wide areas of low-risk or uninhabited cells, which are hard to reach within the same response time as cells in the same category near urban areas. Establishing an ambulance base would not be expedient due to the low number of missions, and even a voluntary first responder scheme is often impossible.

To improve and standardise collection of key figures in EMS, the Ministry of Social Affairs and Health and assigned the FinnHEMS Research and Development Unit the task of developing a new method for nationwide EMS response time data analysis.

The aim of this study was to describe the risk category renewal process and analyse possible differences in mission profiles between the risk category areas.

## Methods

An existing countrywide 1-km^2^ hexagon-shaped cell grid was chosen as the basis for the new nationwide grid. EMS mission data have been collected for statistical and scientific purposes for several years by using the grid, and maintaining comparability over time was considered essential. The grid also covers the autonomous province of Åland and territorial waters, but these areas were excluded from the analysis. The map projection used in the grid is ETRS-TM35FIN, which is a national representation of European Terrestrial Reference System 1989 [6].

In theory, the previous method based on linear regression predictions of missions for each cell could have been improved by applying more advanced statistical models, zero-inflated Poisson or negative binomial models [7]. However, neither of these models substantially facilitates gaining more precise predictions than the previous one. The amount of 1-km^2^ cells with zero missions is overwhelming. Bayesian methods were also considered, but due to their complexity and the need for a high level of expertise, they offer little help in everyday administrative work.

A more straightforward approach based on the Finnish Environment Institute’s (FEI) national definition of urban/rural areas was chosen as the basis for the new classification. FEI updates urban area maps yearly, and they are published as open data according to the INSPIRE directive [8]. In Finland, an urban area is defined as a unified geographical area with a minimum of 200 inhabitants and less than 50 m between buildings. The other Nordic countries have similar urban area definition, with only minor country-specific adjustments [9].

In Finland, public address registers often point addresses to the nearest public road, in other words, mailbox location. In a rural area this may be hundreds of metres away from the building. To get a more accurate location, the building ID of each person’s home address in the Population Information System and the building coordinates from the Planning Permission Register were combined. No other information from these national registers was used in this study. Both national registers are maintained by the Population Register Centre, and the data are available for official or scientific use. Persons without a known address or with a secret address for security reasons were excluded. We excluded from the building permit register a handful of buildings with obviously false locations outside the country’s borders. With this method, 5,375,664 inhabitants (97.7% of population) were located.

**Fig. 1:**
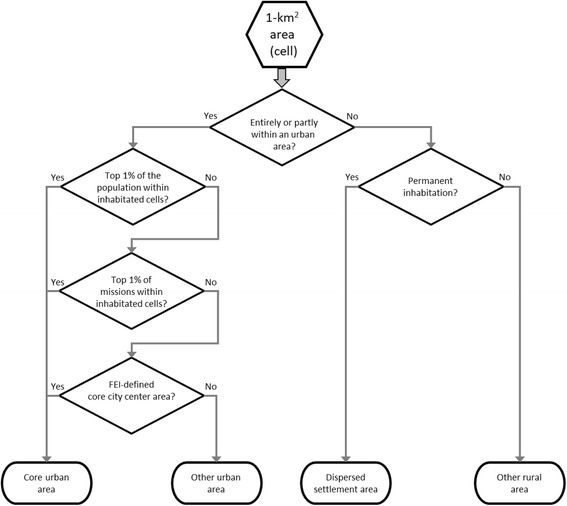
Flowchart of the new risk classification for each 1-km^2^ cell in Finland

Most urban areas are still quite sparsely populated. Thus, we developed the concept of “core urban area” to identify the most urban centres of major cities. A core urban cell is defined by at least one of the following criteria: they are 1) within the top 1% of a HD’s urban area inhabited cells based on population or 2) missions, respectively, or 3) within FEI’s designated core city centre area. As FEI publishes these core centre maps irregularly, the population and mission density criteria were added to the scheme. Rural areas were divided into permanently inhabited and uninhabited cells (Fig. 1).

An additional thematic map shows an example of risk categories (Additional file 1). To evaluate the new risk categorisation, we compared EMS mission profiles between categories by calculating risk ratios with confidence intervals for mission types. We acquired EMS mission data from the Emergency Response Center Authority (ERC) for the period from 1 January 2016 to 31 December 2016. Inter-hospital transportations, missions outside Finnish territory or with missing location data were excluded. A total of 706,004 missions met the inclusion criteria.

All included missions were used for descriptive statistics. For risk ratio calculations, the missions were classified into 11 groups based on the dispatch code. For trauma missions, the four most common trauma mechanisms were included and reported separately. Other trauma missions were excluded from this part of the analysis. The classification is based on the physician-staffed pre-hospital service report template [10], with some modifications. Obstetrics and childbirth missions were excluded due to the small number of missions compared with other groups, and infections were not detectable by ERC dispatch codes. The groups include “First Hour Quintet” [11] mission groups, as well.

The data were analysed with RStudio Version 1.1.383, SPSS 24 and MapInfo 16.0.

## Results

There were 393,105 cells within Finland’s borders and territorial waters at the end of the year 2016. Excluding seas and the autonomous province of Åland, there were 344,212 cells within the HDs’ areas.

Each risk class has a characteristic mission profile, with the orientation of the share of most urgent missions increasing as population density decreases.

Finland’s population appears to be very concentrated. Only 98,234 (28.5%) cells had permanent inhabitants. EMS missions are also concentrated along with the population. A total of 51,920 cells (15.1%) had at least one EMS mission per year, and only 1571 cells (0.5%) had over 100 EMS missions per year.

A total of 87.3% of the population lives and 87.5% of the missions took place in core or other urban areas, which cover 4.7% of the HDs’ areas.

Trauma mission incidence per 1000 inhabitants was higher in core urban areas (42.2) than in other urban (24.2) or dispersed settlement areas (24.6). The results were similar for non-trauma missions (134.8, 93.2 and 92.2, respectively). The differences between area types were statistically significant due to the high number of missions, but in practice the difference between other urban and dispersed settlement areas is negligible (Table 2).

**Table 2:** Descriptive statistics according to the new risk categories

	Cells		Population		Missions		Missions /1000 inhab.		
New risk category	N	%	N	%	N	%	all	trauma	non-trauma
Core urban	390	0.1	1,105,518	20.6	196,023	27.8	177.3	42.2	134.8
Other urban	15,921	4.6	3,588,435	66.8	421,584	59.7	117.5	24.2	93.2
Dispersed settlement	82,816	24.1	681,711	12.7	79,648	11.3	116.8	24.6	92.2
Other rural	245,085	71.2	0	0	8723	1.2	–	–	–

**Fig. 2:**
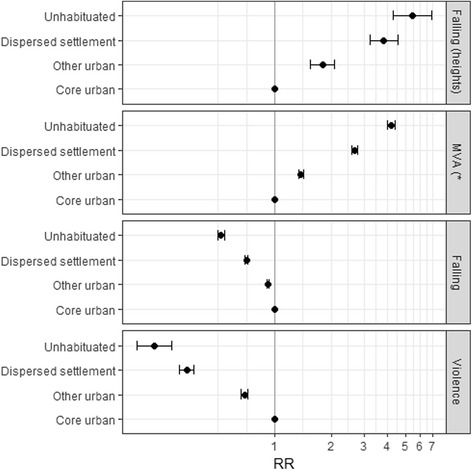
Risk ratios of various trauma missions in the new risk categories. *) MVA = Motor Vehicle Accident

Falling from an over 2-m height and motor vehicle accidents (MVA) were the most common trauma mechanisms in uninhabited and dispersed settlement areas, while falling on ground level and violence-related traumas dominated in urban areas (Fig. 2).

**Fig. 3:**
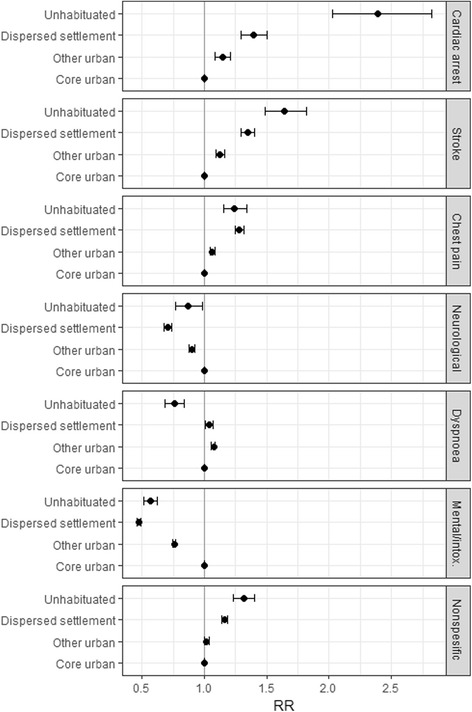
Risk ratios of non-traumatic mission groups in the proposed risk categories

Within non-trauma missions, vascular and cardiac problems were most common in uninhabited and dispersed settlement areas. Especially the share of cardiac arrest missions was noticeably high within uninhabited areas, along with chest pain and stroke. Psychiatric problems and intoxication along with neurological problems (excl. stroke) are more common in urban and core urban areas (Fig. 3).

## Discussion

The main findings of this study were that 1) each of the proposed risk categories has a characteristic mission profile, 2) high-energy trauma missions and cardiac problems were more common in rural and uninhabited cells, while violence, intoxication and non-specific problems dominated in urban areas and 3) the grid-based data collection enables analysis of EMS mission data independently of municipality, hospital district or other administrative boundaries.

We found that categorising areas is a complex issue. To be useful in everyday life, categories should be easily determined, internally consistent, comparable between administrative areas and reflective of the layman’s assumption about availability of EMS services. Categorisation in the presented solution was based on an existing and widely used urban-rural classification. From a national perspective, each category has a typical mission profile. In rural areas, vascular problems, falling from heights and motor vehicle accidents are more common, whereas violence, mental problems and intoxication dominate in urban areas.

The high share of cardiac arrest missions in uninhabited areas was an unexpected finding, although other high-risk mission types also appear to be more common in sparsely populated areas. Even though the number of missions in rural areas is quite small, the higher probability of high-risk missions should be considered when planning EMS. For example, new methods for improving access to early defibrillation in rural areas should be developed.

The grid-based approach is not unique, but it is generally used e.g. in epidemiological studies utilizing geographic analysis [12, 13]. Using the grid may cause inaccuracy and fluctuation in statistics when administrational and cell boundaries are not convergent. Thus, for statistical purposes it is necessary to collect statistical data by using administrative areas as well. Grid-based data collection is useful for scientific and service planning purposes, but not always suitable for political decision-making.

The method described in this article is not depend on grid projection, and comparable results can be achieved if each cell area stands 1 km^2^. Tools needed for this analysis are the grid file, mission coordinates and population home addresses location. If similar area division would be used, most likely the results would have similar trends. However, it is true that Finland has quite low population density even in the capital region, and this might weaken generalizability of the results.

The future will show whether the newly developed method will solve the methodological flaws observed in the previous (2011) legislation and risk category system.

## Conclusion

The proposed 1-km^2^ grid-based data collection and area classification appears to be a useful method for evaluating EMS demand and availability in different parts of the country for statistical purposes. Due to a common rural/urban area definition, the method might be usable for comparison between the Nordic countries.

## Additional file

Additional file 1: Thematic map of risk categories. An example of the risk categories around city of Jyväskylä, Central Finland, based on 2016 EMS missions and population in Jan. 2017. (PDF 7832 kb)

## Abbreviations

EMS: Emergency medical service
ERC: Emergency response center
FEI: Finnish environment institute
HD: Hospital district
HEMS: Helicopter emergency medical service
MVA: Motor vehicle accident

## References

[1] Wankhade P (2011). Performance measurement and the UK emergency ambulance service. International Journal of Public Sector Management.

[2] Bevan G, Hamblin R (2009). Hitting and missing targets by ambulance Services for Emergency Calls: effects of different Systems of Performance Measurement within the UK. J. R. Stat. Soc.: Series A (Statistics in Society).

[3] Fitch & Associates, LLC. Performance evaluation of Nova Scotia emergency health services: consultant report. In: Canadian Research Index. Ann Arbor: ProQuest Micromedia; 2001.

[4] Lindström V, Pappinen J, Falk A, Castrén M (2011). Implementation of a new emergency medical communication Centre organization in Finland--an evaluation, with performance indicators. Scand. J. Trauma Resusc. Emerg. Med.

[5] Laukkanen-Nevala P, Olkinuora A, Riihimäki J, Pappinen J, Silfvast T, Virkkunen I, et al. Valtakunnallinen selvitys ensihoitopalvelun toiminnasta.&nbsp;Sosiaali- ja terveysministeriön raportteja ja muistioita 2016:67. Helsinki: Sosiaali- ja terveysministeriö; 2016.

[6] European Union legislation. COMMISSION REGULATION (EU) No 1089/2010 of 23 November 2010 implementing Directive 2007/2/EC of the European Parliament and of the Council as regards interoperability of spatial data sets and services, (23 Nov, 2010).

[7] Cameron AC, Trivedi PK. Regression analysis of count data. 1. Publ., 7. Print. Ed. Cambridge [u.a.]: Cambridge Univ. Press; 2008.

[8] European Union legislation. Directive 2007/2/EC of the European Parliament and of the Council of 14 March 2007 establishing an Infrastructure for Spatial Information in the European Community (INSPIRE). (14th March, 2007).

[9] Wadman M (2009). Sveriges officiella statistik. Tätorter 1960–2005.

[10] Krüger AJ, Lockey D, Kurola J, Di Bartolomeo S, Castrén M, Mikkelsen S (2011). A consensus-based template for documenting and reporting in physician-staffed pre-hospital services. Scand. J. Trauma Resusc. Emerg. Med.

[11] Krafft T, García Castrillo-Riesgo L, Edwards S, Fischer M, Overton J, Robertson-Steel I (2003). European emergency data project (EED project): EMS data-based health surveillance system. Eur J Pub Health.

[12] Baker DM, Valleron A (2014). An open source software for fast grid-based data-mining in spatial epidemiology (FGBASE). Int J Health Geogr.

[13] Ersbøll AK, Kjærulff TM, Bihrmann K, Schipperijn J, Gislason G, Larsen ML (2016). Geographical variation in a fatal outcome of acute myocardial infarction and association with contact to a general practitioner. Spatial and Spatio-temporal Epidemiology.

