# Supplementary material for: Development and implementation of a geographical area categorisation method with targeted performance indicators for nationwide EMS in Finland
Source: Scand J Trauma Resusc Emerg Med. 2018 May 15;26:41. doi: 10.1186/s13049-018-0506-1 (PMC5952514; doi:10.1186/s13049-018-0506-1)
Supplement: Supplementary file 1 — Thematic map of risk categories. An example of the risk categories around city of Jyväskylä, Central Finland, based on 2016 EMS missions and population in Jan. 2017. (PDF 7832 kb) [file 13049_2018_506_MOESM1_ESM.pdf]

Additional file 1

Thematic map of risk categories

Example of risk categories around city of Jyväskylä, Central FInland based on 2016 EMS missions and population in Jan. 2017.

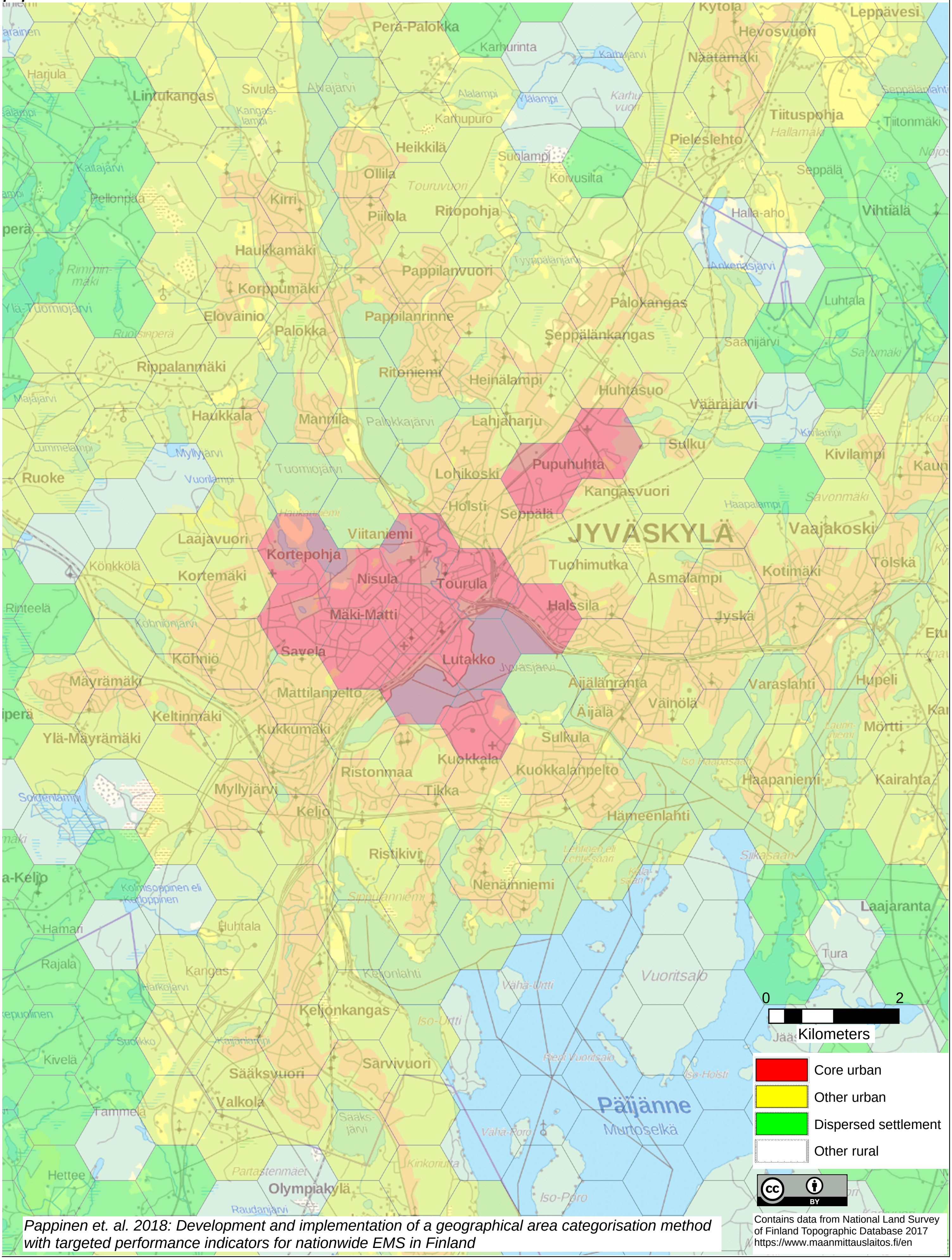

Pappinen et. al. 2018: Development and implementation of a geographical area categorisation method with targeted performance indicators for nationwide EMS in Finland

Contains data from National Land Survey of Finland Topographic Database 2017
https://www.maanmittauslaitos.fi/en
